# Methotrexate as an oral corticosteroid-sparing agent in severe asthma: the emergence of a responder asthma endotype

**DOI:** 10.3402/ecrj.v1.25037

**Published:** 2014-11-14

**Authors:** Malene Knarborg, Ole Hilberg, Hans-Jürgen Hoffmann, Ronald Dahl

**Affiliations:** 1 Department of Pulmonary Medicine and Allergology, Aarhus University Hospital, Aarhus, Denmark; 2 Department of Clinical Medicine, Aarhus University Hospital, Aarhus, Denmark; 3 Department of Dermatology and Allergy Centre, Odense University Hospital, Odense, Denmark

**Keywords:** corticosteroid-dependent, corticosteroids, prednisolone, side effects, dose reduction

## Abstract

**Background:**

Sustained use of oral corticosteroids is associated with significant side effects. It is therefore of interest to find a corticosteroid-sparing agent. In two meta-analyses, methotrexate resulted in a rather small reduction in the oral corticosteroid maintenance dose. We have used methotrexate as an oral corticosteroid-sparing agent in consecutive patients with severe bronchial asthma and find a need for a real-life observational study to evaluate the effect of methotrexate in clinical practice.

**Methods:**

We analyzed the clinical data of 13 oral corticosteroid-dependent asthma patients with a mean prednisolone dose of 15 mg/day for up to 8 years. The diagnosis of asthma based on the clinical history, positive bronchodilator reversibility test, and variable airflow obstruction was secured by bronchial biopsies in all patients. We reviewed the literature and found 12 studies evaluating methotrexate as an oral corticosteroid-sparing agent in severe asthma and calculated the mean daily reduction in mg of prednisolone.

**Results:**

Oral corticosteroids could be reduced in 8/13 patients, 61.5% (mean reduction 9.0 mg/day), and stopped in six of these patients. Five patients had no reduction and remained oral corticosteroid-dependent. Patients with the highest oral corticosteroid doses experienced the greatest reductions. Two patients stopped methotrexate due to side effects. FEV1 remained unaffected by methotrexate treatment and corticosteroid reduction.

**Conclusions:**

Methotrexate has significant oral corticosteroid-sparing effect while maintaining an unaltered asthma control and spirometry. Methotrexate seems an effective oral corticosteroid-sparing agent in a significant proportion of patients with severe asthma. The specific asthma phenotype/endotype that responds needs further study.

Asthma is a chronic inflammatory airway disorder affecting approximately 235 million people worldwide of all ages and ethnic backgrounds (1). The number of asthma patients is estimated to grow by 100 million by 2025 (2, 3). Asthma is associated with reversible airway obstruction, airway hyper responsiveness, and variable airflow obstruction, causing recurrent attacks of breathlessness and wheezing occurring with variable frequency and severity in different patients (2). Approximately 3% of patients with asthma suffer from severe asthma and less than 1% of patients suffer from difficult-to-treat asthma that requires systemic corticosteroid treatment in order to achieve symptom control. Most resources in specialized asthma units are used on patients with severe asthma (4, 5).

According to WHO, treatment-resistant severe asthma is defined as asthma controlled or sometimes uncontrolled by the highest level of recommended treatment (6, 7). In the newly published ERS/ATS guidelines, the definition is more distinct; it is described as ‘Asthma which requires treatment with guidelines suggested medications for GINA steps 4–5 asthma (high dose ICS and LABA or leukotriene modifier/theophylline) for the previous year or systemic CS for ≥50% of the previous year to prevent it from becoming “uncontrolled” or which remains “uncontrolled” despite this therapy’ (8).

The therapeutic options for patients with severe corticosteroid-dependent asthma are limited, and often we have to accept a lesser degree of symptom control. A number of studies point to the use of various interventions with documented effects according to Global Initiative for Asthma (GINA) guidelines.

Anti-IgE treatment with omalizumab (Xolair) has documented effect in patients with severe asthma, frequent exacerbations, and perennial allergy. The treatment is administered subcutaneously every 2–4 weeks depending on body weight and serum total IgE concentration. A cost-benefit analysis concluded that omalizumab is cost-saving in non-smoking patients admitted minimum five times or minimum 20 days per year despite the highest level of asthma care and treatment (9).

Methotrexate is rather infrequently used for ‘treatment-resistant asthma’ and its use is limited by side effects. In 2012, methotrexate was added to the GINA guidelines as a treatment option in selected patients suffering from severe asthma treated at highly specialized asthma units under the supervision of an asthma specialist (4, 5, 10).

A Cochrane review from 2009 found a corticosteroid-sparing effect of methotrexate (11), but the authors expressed some reservation regarding the quality of some of the studies included.

We find a need for a real-life observational study to evaluate the effect of methotrexate as a corticosteroid-sparing agent in the treatment of severe asthma. The aim of this study was therefore in a well-controlled real-life setting at a highly specialized asthma unit to further evaluate if methotrexate was effective as an oral corticosteroid-sparing agent in the treatment of patients with severe oral corticosteroid-dependent asthma.

## Methods

We evaluated all of the 15 oral corticosteroid-dependent patients (10 women and 5 men) with severe asthma that were treated with methotrexate at the Department of Pulmonary Medicine at Aarhus University Hospital, Denmark. The patients were treated with oral corticosteroids, because it was not possible to prevent asthma from becoming uncontrolled without oral corticosteroids despite maximum asthma treatment. Before the addition of methotrexate, the asthma diagnosis was confirmed by a clinical history typical of asthma, long observation period with variable airway obstruction, positive reversibility test with short-acting beta-2-agonist and high-resolution computed tomography (HRCT) scan of the lungs, and the diagnosis was secured by bronchoscopy with deep mucosal bronchial biopsies to avoid misdiagnosis. Absence of signs of emphysema and the presence of basement membrane thickening and/or disruption of the epithelium were criteria to support the diagnosis of asthma before initiating treatment with methotrexate as oral corticosteroid-sparing agent.

Contraindications for methotrexate were kidney or liver diseases, and pregnancy. Contraception was mandatory for women and men treated with methotrexate as well as for their partners. There was no age limit. Men and women were included alike. The patient's progress was followed individually for up to 8 years.

A literature search on trials evaluating the oral corticosteroid-sparing effect of methotrexate was done to calculate the mean reduction in daily oral corticosteroid dose in previous studies.

## Literature search

The literature search was conducted in ‘PubMed’ searching the MEDLINE library for articles investigating methotrexate as oral corticosteroid-sparing agent in the treatment of severe asthma in adults. We applied the following search terms ‘Severe asthma’ AND ‘methotrexate’ and searched reference lists in clinical trials and review articles.

We restricted the search to English-language articles. Randomized, placebo-controlled trials and a single 12-month-long prospective observational study were selected for inclusion in the study along with our observational study.

## Statistical analysis

The Wilcoxon signed rank test was used for evaluation of the reduction in prednisolone dose.

## Results

We treated a total of 15 patients with severe asthma receiving prednisolone in addition to maximum inhaled long-acting bronchodilators and corticosteroid. Before the introduction of methotrexate, the oral prednisolone dose necessary to avoid exacerbations and maintain symptom control as good as possible was established. Mean prednisolone dose before methotrexate was 15 mg/day.

The demographic and clinical data of the patients are shown in Table 1. Two women were excluded, because they stopped treatment after only 3 and 4 months, respectively, due to side effects. The final 13 patients in our study (eight women and five men) were followed from 7 months up to 8 years with monthly check-ups. Four patients were ex-smokers, one current smoker and eight non-smokers.

**Table 1:** Demographic and clinical characteristics of the study population

	Mean	SD
Male/female	38%/62%	
Age (years)	50.5	15
Weight (kg)	76	20.6
Height (cm)	166.33	8.7
FEV1 (%) Pre	76	21
FEV1 (%) Post	76	27
Years of asthma	12	12.8

The methotrexate maintenance dose was 15 mg/week in 11 patients, 12.5 mg/week in 1 patient, and 10 mg/week in 1 patient (mean dose 14.6 mg [SD 1.72]). Two patients with an overall methotrexate maintenance dose of 15 mg/week had 20 mg/week for 1 year.

**Fig 1:**
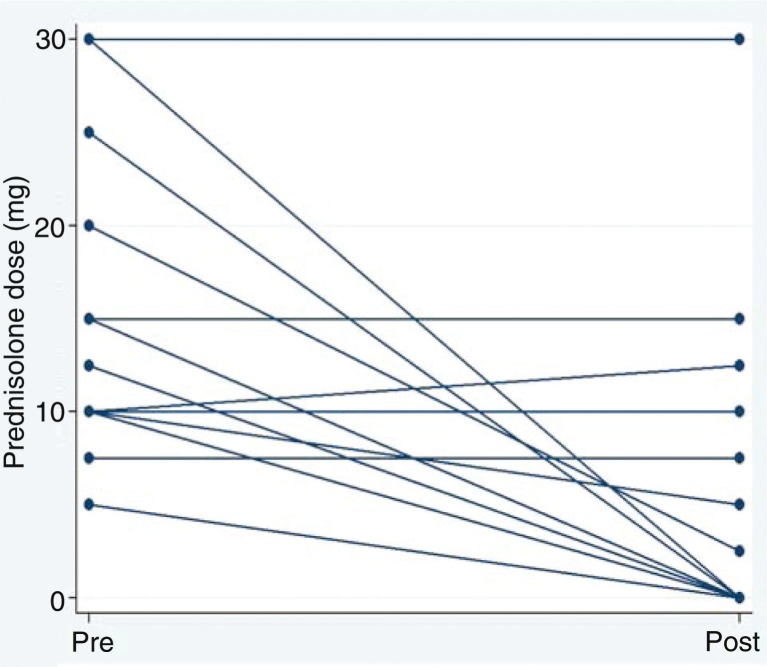
Oral prednisolone dose (mg/day). Pre- and post-introduction of methotrexate in the 13 patients in our study.

Oral prednisolone could be reduced in eight patients (61.5%). Prednisolone dose was tapered at varying intervals depending on an individual medical assessment focusing on the patient's clinical condition. Four patients reduced prednisolone to 0–2.5 mg/day within less than 3 months; three of them stopped prednisolone within 2 months, one reduced from 10 to 2.5 mg/day within 3 months. The four other patients reduced prednisolone over a maximum of 15 months. Before discontinuation of methotrexate, the patients who stopped methotrexate early due to side effects reduced their prednisolone dose from 30 mg/day to 5 and 15 mg/day, respectively. Fig. 1 shows pre- and post-doses of the 13 patients treated for ≥7 months. Prednisolone could be stopped in six patients (46%). Five patients remained prednisolone-dependent despite methotrexate (38.5%). Three patients increased their prednisolone dose despite methotrexate (23%).

The mean reduction in prednisolone in all 13 patients was 9.03 mg/day (SD 10.44) or 58.75% *p*<0.01. The mean prednisolone reduction was 15 mg/day (SD 8.96) or 94% in the eight patients, who were able to reduce the prednisolone dose.

The patients tolerated methotrexate well with mainly minor side effects that ceased on discontinuation of treatment. The side effects in the patients who stopped methotrexate early were one case of cystitis and one case of mouth sores and abdominal pain. Among patients continuing in the study, one had long-term fever and elevated liver enzymes after 1.5 years treatment, one had gastrointestinal side effects, one briefly had slightly elevated liver enzymes and nausea, one had a brief period of nausea, and one patient had skin defects and dysesthesia. One patient died of causes unrelated to methotrexate after being in the study for 2 years and 10 months. No cases of lung fibrosis were observed.

FEV1 remained unaffected by methotrexate and oral prednisolone reduction. Initial mean value of FEV1 was 2.2 L (SD 0.66) or 76% of predicted normal value, and after treatment 2.18 L (SD 0.86) (76.4% predicted).

The literature search identified 11 randomized, placebo-controlled studies, one long-term prospective observational study, and three meta-analyses from 1997, 1998, and 2009 investigating the role of methotrexate as an oral corticosteroid-sparing agent in the treatment of severe asthma.

The first meta-analysis based on 11 randomized, placebo-controlled trials concluded that there was significant corticosteroid-sparing effect of methotrexate with a 4.37 mg/day or 24% reduction in oral corticosteroid (12). The effect was most pronounced when methotrexate was used for more than 24 weeks. The author called for long-term studies to more thoroughly address the effect and side effects of methotrexate.

A second meta-analysis soon followed that included the same studies plus an unpublished trial with negative results. This resulted in a mean reduction of 3.3 mg/day or 18.2% in oral corticosteroid (13). The author also constructed a meta-analysis of side effects and concluded that the modest corticosteroid-sparing effect of methotrexate did not seem to outweigh the disadvantages.

The third meta-analysis was published as a Cochrane review in 2000 and reprinted in 2009 after further literature search. Only trials of more than 12 week's duration were included (*n*=10) (11).

Two studies of 12 months have since been published: A randomized, placebo-controlled study (14) and a single-center prospective observational study (4). Consistent with our observational study, they found a significant corticosteroid-sparing effect of methotrexate.

**Fig 2:**
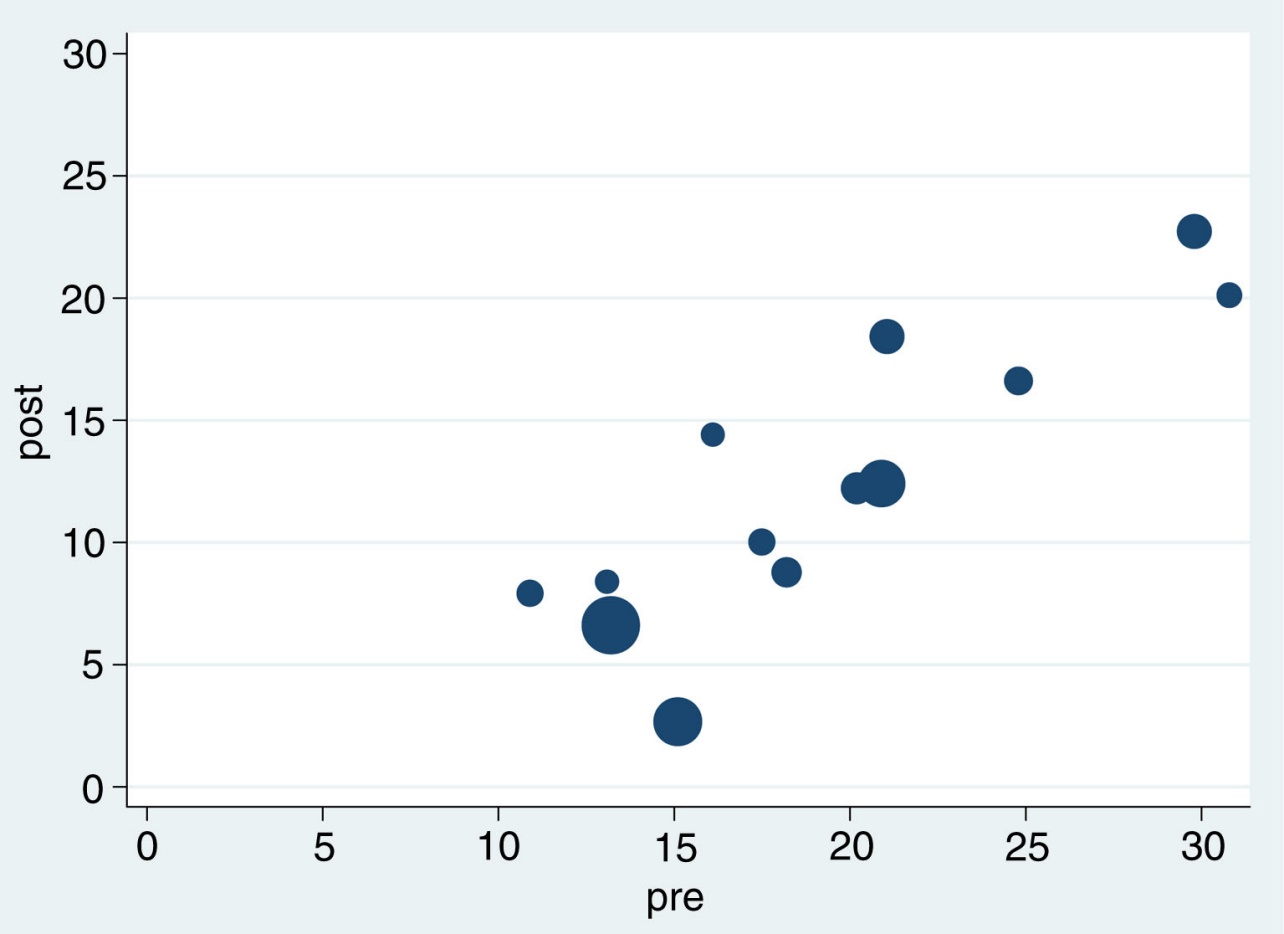
The oral corticosteroid dose reduction in 13 studies with methotrexate as corticosteroid-sparing agent (Table 2). Corticosteroid doses pre and post methotrexate treatment are shown. The size of dot reflects the number of patients in each study. The regression coefficient is 0.35 and equivalent to a mean reduction of 35%.

In Table 2, the results of our observational study and 12 previous original studies are summarized. The meta-analysis from 1997 included 11 studies (12). One of these evaluated the cumulative dose of oral steroids and was not included in the present evaluation. We did not include the unpublished study included in the second meta-analysis, but found two new studies plus our own. Fig. 2 shows the prednisolone doses pre and post methotrexate treatment in the 13 methotrexate studies listed in Table 2. The regression coefficient is 0.35 equivalent to a mean reduction of 35% (CI=0.27–0.45) demonstrating the greater effect seen in patients receiving the highest corticosteroid doses.

**Table 2:** Reference list of 13 studies evaluating methotrexate as an oral corticosteroid-sparing agent

Author, year	Title	Method	Dosage of MTX	Duration	Patients included; patients complete*d*=*n*	Results; corticosteroid mg/day (SD)	Side effects
Mullarkey et al., 1988 (15)	Methotrexate in the treatment of corticosteroid-dependent asthma: a double-blind crossover study	Oral methotrexate or matched placebo weekly administered in three divided doses 12 h apart in randomized cross-over trial.	15 mg	24 weeks. 2×12 week treatment period.	22 *n*=13	Start dose 24.8 mg (15.3) End 16.6 mg (15). Reduction 8.2 mg or 33.1% Discontinuation in one patient.	Nausea, rash, elevated liver enzymes.
Shiner et al., 1990 (16)	Randomized, double-blind, placebo-controlled trial of methotrexate in steroid-dependent asthma	Randomized parallel trial of oral methotrexate or matched placebo weekly.	15 mg	38 weeks. 24 weeks treatment.	69 *n*=60	Start dose 13.2 mg (5). End 6.6 mg (6) Reduction 6.6 mg or 50%. Discontinuation in six patients.	Elevated liver enzymes in 12 patients. Gastrointestinal symptoms, mainly heartburn, nausea, bloated abdomen and diarrhea.
Dyer et al., 1991 (17)	Methotrexate in the treatment of steroid-dependent asthma	Randomized cross-over study of oral methotrexate or matched placebo weekly.	15 mg	8 months. 2×12 weeks treatment, 4 weeks wash-out period in between	12 *n*=10	Start dose 13.1 mg (5.31). End 8.37 mg (2.96) Reduction 4.73 mg or 36.1%.	Mild side effects including anorexia, alopecia and stomatitis. Resolved with dose reduction.
Erzurum et al., 1991 (18)	Lack of benefit of methotrexate in severe, steroid-dependent asthma: a double-blind, placebo-controlled study	Randomized parallel trial of methotrexate or placebo intramuscularly once weekly with open trial of methotrexate at the conclusion of the double-blind study.	5 mg in week 1. 10 mg in week 2, then 15 mg weekly.	13 weeks. 12 weeks treatment.	19 *n*=17	Start dose 20.2 mg (5). End 12.2 mg (5.12). Reduction 8.0 mg or 39.6%.	Gastrointestinal side effects. Nausea, diarrhea, elevation of transaminases, alopecia. One patient died during follow-up of pneumocystis carinii pneumonia. No significant difference between groups.
Taylor et al., 1993 (19)	Methotrexate in the management of severe steroid dependent asthma	Randomized cross-over trial of oral methotrexate or matched placebo weekly.	7.5 mg week 1 of each period, then 15 mg.	48 weeks. 2×24 weeks treatment.	11 *n*=9	Start dose 16.1 mg (7.5). End 14.4 mg (6.8). Reduction 1.7 mg or 10.6%.	Frequent. Elevated liver enzymes. One case of nausea, vomiting and mild alopecia.
Trigg and Davies, 1993 (20)	Comparison of methotrexate 30 mg per week with placebo in chronic steroid-dependent asthma: a 12-week double-blind cross-over study	Increasing doses of methotrexate or placebo administered in three divided doses 12 h apart in randomized cross-over study.	7.5 mg in week 1. 15 mg in week 2, then 30 mg weekly.	24 weeks. 2×12 week treatment.	18 *n*=12	Start dose 17.5 mg (11.55). End 10 mg (5.3) Reduction 7.5 or 42.9%.	Frequent. Headache, pneumonia, elevated liver enzymes, stomatitis, zoster, cellulitis, pityriasis versicolor. Dose reduction necessary.
Coffey et al., 1994 (21)	The role of methotrexate in the management of steroid-dependent asthma	Prospective randomized cross-over trial of methotrexate or matched placebo in increasing doses.	7.5 mg week 1–2. Increased by 2.5 mg every 2 weeks up to 15 mg weekly.	28–34 weeks. 2×12 weeks treatment.	14 *n*=11	Start dose 30.78 mg (16.25) End 20.1 mg (12.6). Reduction 10.68 mg or 34.7%.	No significant difference between groups. Mainly abdominal pain, nausea and diarrhea. One patient on placebo withdrawn due to alopecia.
Stewart et al., 1994 (22)	Comparison of oral pulse methotrexate with placebo in the treatment of severe glucocorticosteroid-dependent asthma	Randomized cross-over study of oral methotrexate or matched placebo weekly.	7.5 mg first 3 weeks, then 15 mg	33 weeks. 2×12 weeks treatment, 3 weeks wash-out period in between.	24 *n*=21	Start dose 21.05 mg (6). End 18.4 mg (9.9). Reduction 2.65 mg or 12.6%.	Mild side effects. Nausea, headache, upper respiratory tract infections, diarrhea.
Kanzow et al., 1995 (23)	Short-Term Effect of Methotrexate in Severe Steroid-dependent Asthma	Randomized, parallel trial of oral methotrexate or matched placebo weekly.	15 mg	27 weeks. 16 weeks treatment.	24 *n*=21	Start dose 29.8 mg (13.9). End 22.7 mg (13.3). Reduction 7.1 mg or 23.8%.	Slight nausea in two patients.
Hedman et al., 1996 (24)	Controlled trial of methotrexate in patients with severe chronic asthma	Randomized, cross-over trial of oral methotrexate or matched placebo weekly.	15 mg	26 weeks, 2×12 weeks treatment.	13 *n*=12	Start dose 10.9 mg (8.4). End 7.9 (8.1). Reduction 3 mg or 38%.	One patient withdrawn due to nosebleeds. Other side effects were vomiting, nausea, abdominal pain.
Comet et al., 2005 (14)	Benefits of low weekly doses of methotrexate in steroid-dependent asthmatic patients. A double-blind, randomized, placebo-controlled study	Randomized, parallel trial of oral methotrexate or matched placebo weekly.	10 mg	12 months	46 *n*=39	Start dose 17.3 mg (63.39) End 7.8 mg (31.85). Reduction 9.5 mg or 54.8%.	One case of diarrhea, one of bronchospasm, one of unspecific symptoms.
Domingo et al., 2009 (4)	Twelve years’ experience with methotrexate for GINA treatment step 5 asthma patients	Oral methotrexate weekly systematically offered to all who met the inclusion criteria.	10 mg	12 month 91.3±39.5 months	44 *n*=42	Start dose 15.1 mg (53.14). End 2.64 mg (34.67) Reduction 12.46 mg or 82.5%. Discontinuation in 25 patients.	One patient with alopecia. Mild increase in hepatic enzymes in four patients. Normalized after discontinuation of treatment.
Knarborg et al., 2014	Methotrexate as an oral corticosteroid-sparing agent in severe asthma: the emergence of a responder asthma endotype	Patients followed from 7 months up to 8 years adjusting treatment of methotrexate and prednisolone according to condition and side effects.	5–20 mg	From 7 months to 8 years	15 *n*=13	Start dose 15.38 mg (SD 8.34) End 6.35 mg (SD 8.88) Reduction 9.03 mg (SD 10.44) or 58.75%. Discontinuation in six patients (46%).	One case of cystitis, one of long-term fever, one of mouth sores and abdominal pain and one of elevated liver enzymes.

## Safety

Some of the studies found no significant difference in the number and severity of side effects between the methotrexate and the placebo groups (Table 2). Patients should be monitored for the most frequently encountered side effects. Folic acid is given to avoid anemia, gastrointestinal side effects, and alterations in taste and sensations. Oral antihistamines may be of value in cases of abdominal pain.

## Discussion

Our study included consecutive patients with severe asthma receiving prednisolone in addition to maximum doses of inhaled long acting bronchodilators and corticosteroid. The oral corticosteroid-sparing effect of methotrexate was evaluated in patients treated for at least 7 months in this well-controlled real-life study of patients with clinical, HRCT, and biopsy-verified severe asthma. The study showed a significant reduction in prednisolone dose of 9.03 mg/day (SD 10.44) or 58.75% in all 13 patients. The reduction occurred mainly in 8 of the 13 cases and in 6 cases prednisolone was stopped completely. The eight best responding patients had a mean reduction of 15 mg/day (SD 8.96) or 94%. This points to the possibility that two distinct groups of severe asthma exist with respect to methotrexate response, which may translate into a specific asthma endotype that responds to methotrexate.

The results of our study are consistent with the results of the Cochrane review from 2009, where a corticosteroid reduction was found in the parallel trials at 4.06 mg/day or 19.2% and in the cross-over trials at 2.86 mg/day or 14.6% (11).

Overall, the oral corticosteroid-sparing effect of low-dose methotrexate is evident. The effects of methotrexate cannot be predicted at the moment but a specific responder group may exist. One of the characteristics is the need of a corticosteroid dose of 10 mg or more. There is a general tendency for methotrexate to show the best results in patients treated with methotrexate for a longer period of time (11) (Table 2) and for patients with the highest oral corticosteroid doses to experience the greatest reductions (Figs. 1 and 2). Further characterization of the responders and factors predicting the effect of methotrexate will be necessary to substantiate this suggestion.

In our study, four patients reduced oral prednisolone within less than 3 months, while for others it took up to 15 months. There were no predictive factors such as asthma duration, pre-study prednisolone dose or methotrexate dose to determine if and when a reduction would occur.

Ideally we should have evaluated the corticosteroid sparing effect of methotrexate in a placebo-controlled study rather than an observational study; however, the severity of the patients’ illness made it problematic to enroll them in a placebo-controlled study. We found it more appropriate to do a real-life observational study, where we were able to reduce the patients’ corticosteroid dose gradually when adding methotrexate to their treatment, as the patients would have had more side effects, if they had continued on the initial high doses of corticosteroid. In a placebo-controlled study, patients on placebo would most likely not be able to reduce their corticosteroid dose.

Patients were compliant to their regular asthma medications and attempts to improve asthma control with other medications like theophylline, leukotriene receptor antagonist, immunotherapy, azathioprine and others had failed. We do not think it has made a difference to the outcome of the study that we conducted a real-life observational study rather than a placebo-controlled study, but a classical double blind placebo controlled trial would have been more convincing and should be performed in a multicenter study of a well characterized patient population.

The potential positive and negative effects of treatment with methotrexate always have to be considered before its use in a specific patient, especially as treatment for a minimum of 6 months is recommended before it is possible to decide if there is an effect. The most prominent side effects reported in studies with low dose methotrexate are gastrointestinal symptoms such as abdominal cramps, diarrhea and slight nausea, mild increase in hepatic enzymes (which in all the mentioned cases was normalized after discontinuation of treatment), alopecia and stomatitis. One patient in a study (18) died of *Pneumocystis carinii* pneumonia probably related to the methotrexate treatment (18, 25, 26). Late findings of methotrexate-induced side effects demonstrate the importance of continuous caution of side effects when treating patients with methotrexate.

The optimal dose of methotrexate is not yet defined. The dose of methotrexate varies between studies from 5 to 30 mg weekly, but 15 mg weekly appears to be sufficient to have an oral corticosteroid-sparing effect and low enough to avoid most side effects. It is possible that the effective dose depends on the intrinsic characteristics of the individual patient, which have to be characterized together with possible responder parameters.

Intensive monitoring and follow-up of patients with severe asthma seems to have a corticosteroid-sparing effect per se given that several studies find dose reduction even in the placebo arm of the studies (14–18, 20, 21, 24). However, we find it unlikely that the oral corticosteroid-sparing effect is related to a ‘regression toward the mean’ effect as patients were seen as frequently before as after introduction of methotrexate because of the severity of the disease. In addition, the finding that reductions were dependent of initial maintenance oral corticosteroid dose across studies could point toward the presence of a responder population.

## Conclusion

Our observational study with clinical, HRCT, and biopsy-verified severe asthma together with results of previous studies supports the beneficial effect of methotrexate as an oral corticosteroid-sparing agent in the treatment of severe asthma. The side effects from methotrexate are usually mild and transient but may lead to cessation of treatment in a few cases.

Methotrexate was associated with a significant corticosteroid-sparing effect and in some cases oral steroids could be stopped, while FEV1 remained unaffected. Despite the overall positive oral corticosteroid-sparing effect of methotrexate, a group of patients remains that does not benefit from the use of methotrexate. The specific asthma phenotype/endotype that responds needs further evaluations.
